# Enablers and barriers to secondary prophylaxis for rheumatic fever among Māori aged 14–21 in New Zealand: a framework method study

**DOI:** 10.1186/s12939-017-0700-1

**Published:** 2017-11-17

**Authors:** Hilary Barker, John G. Oetzel, Nina Scott, Michelle Morley, Polly E. Atatoa Carr, Keri Bolton Oetzel

**Affiliations:** 1 University of Auckland, Faculty of Medical and Health Sciences, Private Bag 92019, Auckland, 1142 New Zealand; 2 Waikato Management School, University of Waikato, Private Bag 3105, Hamilton, 3240 New Zealand; 3 Waikato District Health Board, Pembroke Street, Private Bag 3200, Hamilton, 3240 New Zealand; 4 Independent Contractor, 21 Opotoru Rd, Raglan, 3225 New Zealand

**Keywords:** Acute rheumatic fever, Enabler and barriers to secondary prophylaxis, Health inequities, Māori

## Abstract

**Background:**

Acute rheumatic fever (ARF) rates have declined to near zero in nearly all developed countries. However, in New Zealand rates have not declined since the 1980s. Further, ARF diagnoses in New Zealand are inequitably distributed--occurring almost exclusively in Māori (the indigenous population) and Pacific children--with very low rates in the majority New Zealand European population. With ARF diagnosis, secondary prophylaxis is key to prevent recurrence. The purpose of this study was to identify the perceived enablers and barriers to secondary recurrence prophylaxis following ARF for Māori patients aged 14–21.

**Methods:**

This study took a systems approach, was informed by patient voice and used a framework method to explore potential barriers and enablers to ongoing adherence with monthly antibiotic injections for secondary prophylaxis. Qualitative interviews were conducted with 19 Māori ARF patients receiving recurrence prophylaxis in the Waikato District Health Board region. Participants included those fully adherent to treatment, those with intermittent adherence or those who had been “lost to follow-up.”

**Results:**

Barriers and enablers were presented around three factors: system (including access/resources), relational and individual. Access and resources included district nurses coming to patients as an enabler and lack of income and time off work as barriers. Relational characteristics included support from family and friends as enablers and district nurse communication as predominantly a positive although not enabling factor. Individual characteristics included understanding, personal responsibility and fear/pain of injections.

**Conclusion:**

This detailed exploration of barriers and enablers for ongoing secondary prophylaxis provides important new information for the prevention of recurrent ARF. Among other considerations, a national register, innovative engagement with youth and their families and a comprehensive pain management programme are likely to improve adherence to ongoing secondary prophylaxis and reduce the burden of RHD for New Zealand individuals, families and health system.

## Background

Acute rheumatic fever (ARF) rates have declined to near zero in nearly all developed countries. However, in New Zealand rates have not declined since the 1980s. ARF diagnoses in New Zealand are inequitably distributed--occurring almost exclusively in Māori (the indigenous population) and Pacific children or adolescents [1–4]. A recent review of ARF epidemiology in New Zealand found that more than 90% of cases are diagnosed in Māori or Pacific children/adolescents, with Māori and Pacific approximately 30–40 times (respectively) more likely to be diagnosed with ARF than the majority European/Other population [5]. In the 2013 Census only 23% of the New Zealand child population (aged 14 years and younger) were identified as Māori and 12% within the broad Pacific ethnic group [6]. In 2015, there were 112 hospitalizations for new cases of ARF [7].

While the specific aetiological risks for ARF remain under consideration, cases are associated with poverty and poor housing, including crowded living conditions, under-insulation and inadequate heating and should be considered within the New Zealand health context [2, 7]. New Zealand’s stagnant rates of ARF are set against a background of a publically-funded health system that provides access to healthcare for all citizens/permanent residents including full coverage of secondary prophylaxis with no out-of-pocket expenses and rising child poverty and increasing rates of hospitalisation for diseases of poverty, particularly for Māori and Pacific children [5]. Institutionalised racism has been described as inaction in the face of need [8]. New Zealand’s ongoing and increasing child poverty and housing crises, with resulting health impacts point to a tragic lack of adequate preventative and protective action on multiple levels and in multiple policy arenas. Institutionalised racism then can be in be considered a fundamental driver underlying unacceptably and consistently high ARF rates amongst Māori and Pacific populations in New Zealand over many decades [9–11].

Developing from an autoimmune reaction to untreated group A streptococcal pharyngitis, ARF results in inflammation and swelling of the heart, joints, brain and skin. The inflammation of the heart can lead to rheumatic heart disease (RHD), where heart valves are scarred and may need to be replaced [7]. RHD can have severe impact on patients, their families and communities, may result in premature death in adults and is the primary cause of morbidity (e.g., carditits) and mortality for people who have had ARF [12–14].

In New Zealand, as in most countries, individuals who have had a diagnosed episode of ARF (or documented RHD) are placed in a secondary prophylaxis programme where they receive the continuous administration of antibiotics (most commonly intramuscular injections of benzathine penicillin G or ‘bicillin’) [7]. This is to prevent further infection with group A streptococci and hence the development of recurrent attacks of ARF; recurrence is much more likely to result in RHD and irreversible heart damage. Antibiotic prophylaxis is the most cost-effective way to reduce morbidity and mortality, and this secondary prophylaxis also reduces the severity of RHD [15–18]. Current New Zealand guidelines for the effective secondary prevention of ARF state that bicillin should be administered every 28 days (or 21 days for those with a proven recurrence on 28-day regimen) for the following duration: a minimum of 10 years or until age 21 (whichever is longer) for those with no/mild RHD; at least until age 30 (for those with moderate RHD); up to the age of 40 (for those with severe RHD) [19].

As with treatment for most chronic diseases, adherence remains a challenge; placing children and adolescents who do not achieve consistent adherence at high risk of ARF recurrence and/or RHD [15, 20, 21]. According to unpublished analysis of the patient registry in the region for the current study, the non-adherence rate is approximately 25–30% with most of these cases in the 14–21 year age range. These data are consistent with literature about children and adolescent adherence to treatment for chronic illnesses including the challenge of reaching adolescents who are seeking and asserting independence and their own identity [22, 23]. Prior research has identified a number of factors contributing to adherence of secondary prophylaxis for ARF in developing countries and underserved populations in developed nations (e.g., Aboriginal and Torres Strait Islanders in Australia) [15, 20, 21, 24–29]. Although they have not been organised previously in this manner, there are four key factors: health system, access/resources, relational and individual.

One key feature of the health system is having a register of ARF patients. Health systems with a register and follow-up (e.g., reminder system) have higher levels of adherences than those without a register [20, 26, 28, 30]. A recent review also identified the importance of having enhanced health literacy programmes about the need for injections and the need to have dedicated staff to administer the injections--particularly in community settings [28]. Finally, not having healthcare insurance for the injections is a barrier to adherence in some countries [15].

Access/resources refer to the (lack of) assets that inhibit or facilitate adherence. A major barrier in many studies is the lack of access to treatment for patients exacerbated by living rurally or remotely [21, 24, 29, 31]. Another resource is time off work to get the injections [24]. Further, the household circumstances of patients and their families is another factor that influences treatment adherence; those who commonly changed their place of residence were at greater risk of nonadherence [16]. Finally, some studies found a link between low-income status and poor adherence [25, 32] as well as a link with higher levels of formal education and adherence [21].

Relational characteristics refer to the enablers and barriers within one’s social/cultural network and also the relationship with the healthcare provider. A positive relationship between patients and their healthcare provider is an enabler of adherence [29, 31]. However, as ARF is experienced by marginalised and/or ethnic minority populations across the world, part of the challenge of positive patient-provider relationships is non-biased cross-cultural communication. In fact, some suggest that inadequate attention by providers to cross-cultural issues results in poor communication that has negative implications for adherence [31]. The New Zealand Health Survey includes questions on experience of racism and Māori have reported to experience personal racism in the health setting and this experience of racism is linked to a negative impact on self-reported health [33]. Hence, personal racism from healthcare staff against Māori ARF patients may be a barrier to care in New Zealand. Finally, support from family and friends was found to be an enabler for treatment adherence as this provided practical help as well as encouragement for patients to get their injections regularly [29]. However, some patients identified that their family was sometimes tired of having to support their access to treatment [28, 29].

Individual characteristics include fear/pain of injections and understanding. Injection pain is a major reason for patients missing injections [21, 24, 30, 32, 34]. Fear of the injections arises from repeated painful procedures, and may develop into a phobia of needles if pain is not properly managed [34]. Therefore, pain also fits under the system factor--as the differences in the experience of pain and how much pain becomes a barrier to adherence is influenced by the way that the pain is managed and in the system (like staff training and policies), and by individual staff--rather than being just due to differences in the characteristics of individual patients. There are various techniques for reducing pain that may be supported by the health system or used by a nurse such as applying manual pressure to the injection site, using a ‘Buzzy’ vibrating cold pack and adding the analgesic lidocaine to the injected penicillin [32, 34].

Past research illustrates that patients’ self-reported understanding of both the disease and the importance of secondary prophylaxis were positive factors for adherence, while a lack of understanding was a negative factor [26, 31]. Similarly, a person who was symptomatic or had experienced a severe form of the disease tended to be more motivated to adhere to treatment as they were more aware of the consequences [15, 35]. This understanding is placed within the context of needing to stay adherent for at least 10 years, which can seem like a very long time especially if the importance of these monthly injections is not fully understood, and if a patient finds injections very painful. Thus, there is a need to constantly reinforce patients’ awareness. Understanding is also not simply an individual characteristic, but rather heavily influenced by the relationship with individual staff and the provider themselves – both individually and in the system [16]. More accessible information and appropriate education was desired by participants in several studies [24, 31].

Prior research on the factors of enablers and barriers for secondary prophylaxis of ARF and RHD has mostly been done in developing nations with a few studies in Australia. There is limited literature in New Zealand on this topic, particularly from adolescent patients’ perspective. Regions of New Zealand where ARF diagnoses are most common (particularly in the North Island) are increasingly establishing the key health system characteristics suggested for enhanced adherence including a formal register, follow-up system, dedicated staff and health education programme [4, 28]. Population ARF rates is a New Zealand Ministry of Health priority target and as of 2014, the Ministry of Health requires quarterly reporting of adherence to secondary prophylaxis. Despite these positive changes, ARF rates remain relatively stable and there has been little focus nationally or regionally on adherence, with low levels of long-term compliance a challenge for many areas [36].

Unique contextual influences for Māori and Pacific children and adolescents are important considerations for improving adherence to prophylaxis. The purpose of this study is framed by the following research question: What are the patient-reported enablers and barriers to secondary prophylaxis for ARF in New Zealand for Māori aged 14–21? The region where this study was conducted (the population served by the Waikato District Health Board) includes a high number of Māori children and adolescents, but a small proportion of Pacific youth. Therefore this study focused on the experience and information provided by Māori participants. This study is important as it provides information that illustrates reasons why secondary prophylaxis rates are not higher especially in a publically-funded health system that has placed significant efforts to reduce ARF and enhance secondary prophylaxis. A qualitative approach is taken as often the voice of youth are not included in research and such perspective is important for health efforts aimed to change the behaviour of youth.

## Method

### Design and participants

The study used a framework method to identify patient perspectives of enablers and barriers to adherence to secondary prophylaxis [37, 38]. This study is part of a larger project to incentivise adherence by providing mobile phones and monthly top ups ($20 for data, texting, and phone calls) for patients who receive their monthly injection (“Top up 4 your Top up”). Participant interviews on barriers and enablers to adherence were done during the first month of the incentives project.

Qualitative interviews were performed with 20 ARF patients from the Waikato District Health Board (DHB) region. Final sample size was determined by theoretical saturation of the themes. Selection criteria for the current study included patients on the Waikato Region ARF Register, aged between 14 to 21 years in 2016 who were consented participants in the wider incentives project (*N* = 78). Patients were purposively sampled to include a range of fully adherent patients (*n* = 7), intermittent (those who often were late or missed an occasional injection, *n* = 9), and lost to follow-up (those who missed multiple injections and were not currently receiving injections; *n* = 4). Patients were also included from different geographical areas of the region to ensure a range of urban and rural living situations. We oversampled intermittent and lost to follow-up patients to ensure their perspectives were included although our list was determined a priori. Final participants included the following details: a) ranged in age from 15 to 21 years (M = 18.2, SD = 2.02); b) 11 male and 9 female; c) 15 who live with their family, 3 who move between family members, and 2 who live on their own; and d) 17 Māori, 1 Cook Island Māori, 1 Niuean- Māori, and 1 New Zealand European as self-reported ethnicities (largely consistent with ethnic distribution on the register). We did not include the European participant in the data analysis given the focus on Māori perspectives. Four original participants selected chose not to participate in the study and they were replaced based on similar adherence status.

An overview of the Waikato DHB region is important to place the findings into context. The Waikato DHB is one of 20 serving New Zealand. It spans a large geographic region (more than 20,000 km^2^) in the central part of the North Island and serves more than 400,000 people. The area is largely rural with one medium-sized metropolitan area where the main hospital is located (Hamilton). A second smaller hospital is located in a rural community in the Eastern part of the region (Thames). Primary health organisations are affiliated with the DHB and provide health services through a network of privately-owned yet publically-funded health clinics.

### Data collection procedures

Participants were initially contacted by their district nurse (DN) to invite them into the larger incentives study. They received an information sheet and signed a consent form or assent form for patients under 16 (parental consent provided). The participants in this current study were contacted via text or call and invited to participate in the interview; they provided verbal consent/assent for the interview. The study protocol received approval from the Northern A Health and Disability Ethics Committee and the Waikato Hospital Māori Research Review Committee. Given the rural nature of the region, all interviews were conducted by telephone in English (the first language of participants) and averaged around 20 min. The interviews were conducted by a third-year medical student of New Zealand European ethnicity and were recorded with permission of the participants. The interviewer was trained by two of the research team members (one Māori researcher) in conducting research interviews and also working with Māori patients. She was employed as a summer scholarship student designed to expose medical students to research, particularly involving health inequities.

The interview guide consisted of five categories of open-ended exploratory questions. The first category explored when participants were first diagnosed with ARF. Questions also included those about the initial education received and the experience of their first prophylaxis injections. The second category explored current perceptions about the prophylaxis injections. The third category included questions about barriers to treatment adherence, such as the hardest aspect of the treatment, family involvement and experiences with healthcare providers. The fourth category included questions on what made it easy for participants to get and adhere to treatment. The fifth category requested suggestions for making treatment easier.

### Data analysis

Interview recordings were transcribed and then analysed using framework analysis [37, 38]. Themes within the framework were identified based on three criteria: recurrence, repetition, and forcefulness [39]. The process began with reading of transcripts and identifying initial codes for themes using the multi-factor framework presented in the background section as a loose categorisation scheme. Initial codes were then narrowed to the core themes by identifying similarities and differences in the initial codes. Analysis was completed by the interviewer and confirmed by other research team members, including independent researchers and clinical/population health and Māori health experts. Differences in themes were discussed among the team members until agreement was reached. Analysis included reflexive processes to reflect on power and positionality relative to participants. In particular, this reflexivity led to consideration of larger socio-health inequities in interpreting findings even though participants did not directly identify socio-structural issues including institutional racism in their experiences.

## Results

The findings are organised around the factors presented in the background: access/resources, relational and individual. Health system characteristics are integrated with access. Pseudonyms are used when names are provided with direct quotes.

### Access/resources

In the Waikato DHB region, bicillin injections are delivered in the community, with most patients receiving injections from community DNs at their homes or at school or at a local clinic. This flexible arrangement is a key enabler for adherence. Jerome, a 21-year old male living with his mom, dad and siblings, was lost to follow-up at the time of the study. He talked about past times when the flexibility helped him stay adherent. Most of his injections are received at his house. He worked night shifts, and before the current arrangement he had to go into a clinic to receive his prophylaxis, which was not as easy for him: “I'm on night shift at work and then if they want me to go in (to the clinic) I have to go early and I'll be tired for work. They usually just come out here and do it around lunchtime, around the time I'm waking up.” This flexibility is also important for Chris, 17-year old male, who says having the injections at home is, “The only way I could get it really, because I’m working.” Chris lives with his family was current with his injections despite a busy work schedule because of this flexibility of the DN coming to his home.

Despite this flexibility in administration of injections, some participants experienced a lack of access or resources that created intermittent or lack of adherence. Communication between DNs and patients for appointments was most commonly made by phone, with messages left. Participants commonly described that they could not reply because they did not have phone credit. Jerome, who noted the flexibility in where injections were received helped him stay adherent in the past, was currently lost to follow-up due to a different work schedule and because he did not have a way to contact his DN to make an appointment as he did not have money to pay for phone usage. He also admitted that his lack of adherence was also due to his lack of personal motivation. Some participants also described frequently moving homes and not having needed resources to maintain adherence. For example, Tipene, a 19-year old male living on his own, was lost to follow up for some time. In the past, DNs would come to his home. However, he had moved house and he said he had to go to the hospital to receive injections. This was not feasible as “I don’t have a car, I’ve never lived with someone who has had a car.” He also was not able to text or phone his DN as he did not have credit. He felt the new phone and top-up would help him stay adherent.

Some patients have difficulties due to work conditions or other situations in their lives that limit access to injections. The need to work was an inhibitor for Abel, who is intermittent, 19-year male and living with his family: “Sundays is the only day they can get me in. I work six days a week, and usually on Sundays I'm out running errands because I haven't done them in the week. So, I'm busy as.” He works construction so his work site is not consistent further adding to the complications and this made it difficult to access his injections. Winton a 20-year old male was lost to follow up at the time of his study. He lives on his own, working toward a qualification through study and was in the juvenile system at the time of his initial diagnosis. He had intermittent contact with his family in the past although had current contact with them at the time of the study. He explained his non-adherence to several factors including changing addresses/unstable living situation (past and current), pain and some personal issues he would not elaborate on: “I'm currently changing address. I'm thinking if I get my… because I'm due for my jab, I was due for yesterday but I was out of town on a course camp [studying for his qualification], but I need to get it over and done with. But I'm just really too scared to get it aye, it just scares me because the pain and stuff that I get it's unexplainable…. I'm still like going through a hard time right now.” In sum, these examples illustrate the limitations in resources or access features that serve as barriers to medication adherence in this sample.

### Relational

The support of whānau (extended family members) was an enabler for most patients interviewed. Family members, and particularly parents, provided practical support for patients receiving injections by reminding patients that their injections were due. Family members also motivated and encouraged participants to get injections. For example, Maddison, a 15-year old female who is fully adherent and living with her mum and two brothers said, “Mum’s always like, ‘When are you due? We need to go now.’ ….because I always forget about it.” Some participants described that they could share the difficulties of getting injections with family members, who could empathise and support them. Anika, a 16-year old female who is fully adherent and living with her dad, uncle, and two cousins, said, “One time I told my cousins that I get an injection every month and they're like, ‘Oh yeah, like a flu jab.’ I said ‘No the needle is way bigger’. I showed them the needle and they were like, ‘Oh my god.’” She noted that when they realised the pain and difficulties that she goes through she felt supported. Family members were also helpful for educating participants. Some participants said their parents likely knew more about RF than they did, and that discussions with their parents on this topic were helpful for their understanding. Hohepa, a 16-year old male currently intermittent, and living with his dad, grandmother, and brother, noted, “I can understand them [dad and grandmother] better than the doctors because it was just way easier.” He has good support from his family and he attributes his intermittent adherence to lack of phone credit inhibiting his contact with the DN. This demonstrates how support is not a sufficient condition for adherence for some patients.

In contrast, a few participants did not have any of this type of support in their whānau. Some patients noted they did not talk with their parents about ARF. For Ngaire, a 17-year old female currently intermittent and living with her parents and siblings, this silence about ARF when she first was diagnosed was, “Quite overwhelming” as she did not really understanding what was going on. This lack of discussion led her to not being confident in her knowledge of ARF. She explained that the DNs have helped her be aware and she only misses injections when she travels. William, a 16-year old male currently intermittent living with dad and two sisters stated about his family: “They still don’t know what rheumatic fever means.” He usually gets his injections mainly because his girlfriend insists he does so and is currently intermittent as is away from home (and out of the DHB region); although he noted he is able to get an injections from other region’s DNs if he contacts them. Winton, 20-year old male currently lost to follow and described above noted his father did not understand ARF: “My dad was telling me that it runs in my family.” These barriers were not necessarily the reason patients were not getting injections; rather, it illustrates the complexity of the social relations and is an additional barrier for some ARF patients to overcome. Interestingly all of the patients who described some family barriers were intermittent or lost to follow up at the time of the study.

Further relational enablers and barriers described by participants were support provided by friends. A few patients expressed that they had support from their peers, including friends who also had ARF. For instance, when Jerome, 21-year old male currently lost to follow up, was first diagnosed with ARF he knew a number of people also with ARF, “We used to get our jabs kind of around the same time, so I kind of did know a few people and theirs was a bit worse than mine. We always used to ask each other, have you been for your jab yet?” This speaks to the importance of support although his support occurred years before the current interview and as noted previously, he is working odd hours which is serving as the primary barrier to getting his injections. Several participants explained that it was nice knowing there were other youth going through the same thing they were; Tipene said, “Yeah, I like hearing their stories.” In contrast, some participants mentioned that they wished their work colleagues or school friends knew more about ARF although they were fully adherent and had good family support. For example, Chris exclaimed, “It would make it easier, just to help them understand my situation a bit.” Anika noted that her friends do not understand her and why she has to be careful to avoid exposure to group A streptococcus; “At school they give me leftovers of foods and I’m like, ‘I can't get a strep throat’ and they’ll be like, ‘what’s the big deal?’ These participants noted these relationships as their own personal barriers, although they were not deterrents to remaining adherent because of strong family support and DN flexibility in administering injections.

A final relational influence described was the interactions with the DNs who administer the monthly injections. Most participants said that their nurse was, “Friendly,” “Really nice” and “Cool.” However, when asked if this encouraged them to get their injections, patients said it did not have a large effect. The DN-patient relationship may not have appeared to have directly increased adherence, but was still stated as positive by most patients which may have helped to ease patients fear about injections. In contrast, a few participants expressed that negative interactions with their DNs had put them off getting injections. One example of this included a negative perception of a DN, which led to a patient refusing the injection on the day it was due and delaying the injection for a day with a different DN. For Hohepa, a 16-year old male currently intermittent noted, “Some of them just sounded a bit unsure and I was like, ‘No I'm not doing it with you’. They were like, ‘Is it here or here? You tell me.’ and I was just getting pissed off and I said ‘No I'm not doing it today.’” Karena, a 16-year old female living with her family and currently intermittent was put off getting injections for a while because she was frustrated with the way her nurse was giving her injections and how she spoke to her: “There was one nurse that I didn't really like because she had done it too rough and fast. I actually stopped getting them because I was frustrated… I just hate how they were just like, ‘Think about your future’ and all that.” These relationships illustrated a lack of trust in the DN and his/her ability to administer the injections well, and were direct barriers to adherence.

### Individual

Understanding of ARF was a key enabler or barrier depending on the participants’ specific understanding. Participants who understand ARF and its seriousness had a greater appreciation of why another episode of ARF could be detrimental to their health, and therefore reported being likely to adhere to injections. When asked what they had been told about ARF, participants in the fully adherent group tended to give accurate and concise answers. Anika (16 and adherent) said, “I was told that two of my valves weren't working properly and blood wasn't pumping through my body properly.” Hemi, a 16-year old female living with her parents and brother and also fully adherent, said that ARF, “Starts off from the strep throat and then it's on your valves. Blood leaks back in and it makes extra work on the heart.” The link made to the heart not working properly was described as a key reason why ARF is a very serious condition and injections are needed. In contrast, intermittent and lost to follow-up participants tended to report they did not know much about ARF or could not remember. Jerome (21 and lost to follow up) stated that he had only been told the basics about ARF, and then was just given pamphlets to read. When asked if these were helpful he responded, “I didn’t even read them at all. But I think if I had, I would've known the extent of how bad things could have been. But since I didn't read it, I kind of just brushed it off like, ‘It'll be alright.’”.

Additionally, understanding of the purpose of the injections every month was also an enabler or barrier depending on the participant. For example, several intermittent or lost to follow-up participants did not know why they needed injections every month even though they might have known some information about ARF. For example, Adrian a 15-year old male who is intermittent and moves around a lot and lived with different family members noted, “It helps keep away the bugs. Yeah that's all I know.” Despite this moderate knowledge, he knew he had to have it every 28 days and was late with some injections because of lack of phone credit to make appointments or just being out “doing kid things.” Winton, 20 and lost of follow up noted, “At the time I just thought they wanted to give me injections just for the sake of it.” His lack of knowledge is likely attributed to his being in the juvenile system at the initial diagnosis. In contrast, fully adherent patients had the strongest understanding. For example, Shelby, a 20-year old female living with mom and two brothers explained, “They (DNs) told me that there is a chance I could get exposed to rheumatic fever again and it would be worse than it was before.” Some intermittent participants also noted the importance of injections as a reason for deciding to get their injection again. Hohepa, 16-year old male currently intermittent, said that he had been told by DNs, “Just that it could cause me to get rheumatic fever again. That I might even have to have surgery and stuff; it might be that bad.”

Additional factors described as individual are responsibility and motivation. Participants who reported personal responsibility for their health and those around them were also more likely to be fully adherent. For example, Careen, a 21-year old female living with parents and siblings and adherent, did her own work to investigate ARF which encouraged her to be adherent: “Researched a bit on the Internet to see what caused it and what happens, what was going to happen.” A final aspect of responsibility was driven by fear of significant consequences. Anika (16 and adherent) noted, “To be honest, thinking of having to be going to the hospital again. You might have to have open heart surgery and then that'll make things worse. Like getting it the first time was the worst, so I wouldn't want to go back through that.” The corresponding barrier described was apathy and a lack of motivation as Jerome (21 and lost to follow up) noted, “Oh I just couldn't be bothered. Being so constant and every single month, for however long I had to do it; I was just over it and I couldn't be bothered anymore.” Some intermittent or lost to follow up participants initially were apathetic and then had a change of heart. For example, Rhys, a 20-year old living with parents and two siblings had not been getting injections for some time noted, “I wanted to get them. I asked my mum if she could get a hold of them, and I started getting back into it.”

A final individual factor described was the pain experienced from bicillin injections. Every participant described finding the injections painful at least once. This experience of pain was described as a significant barrier to adherence for some participants, but typically not for those who also described that they were motivated or had a good understanding of the need for the injections. Many participants stated that they were now ‘used to’ the pain or that they did not find it painful anymore. For instance, Chris (17 and adherent) said he found that the injections were, “Just average to me now, I’m used to them” but he, “used to throw tantrums, because I was scared.” He said what made it scary was the needle, and that it was painful. For a number of participants, the pain lasted for a few days after the injection. For Karena (19 and intermittent), “It [the pain] really just lasts for two days on just that side so I just don't sleep on that side for two days.” For some participants, the amount of pain experienced depended on the person giving the injection. Hohepa (16 and intermittent) explained the pain “depends really because I have my favourite nurses…The good ones they're usually sore for a day or two days [the bad ones are longer].” Some participants stated that the reason they have missed injections in the past was because the injections limited their ability to play sport and be as active as they wanted to. For example, Winton (20 and lost of follow up) said, “Everybody loves to do sports and I love my sports and I gave it up for that one particular reason, because I love my sports.” He said the pain from injections had stopped him from playing. Winton noted he was not offered any pain relief and internalised this situation: “I've just gotta get used to it. This is my problem and I'm just gonna have to get used to it.”

## Discussion

ARF, a disease associated with poverty and overcrowding, is not only a poignant illustration of the devastating impact of the unfair distribution of these essential determinants of health by ethnic group, but also of the results of inadequate action to prevent high rates of ARF amongst those most at risk and also to prevent those with ARF from going on to develop RHD [10, 11, 40]. It is imperative that those with ARF are not disadvantaged further by developing RHD, which can result in severe illness, long periods of hospitalisation and premature death [2, 5, 12]. These two diseases impact on quality and length of life for patients and their families. More can and must be done in New Zealand to ensure improved support for those with ARF, so that adherence to secondary prophylaxis is improved and RHD rates can be eliminated [5, 14]. Our research identified multiple small improvements across the system of health care provision.

Our participants reported a variety of relational and individual factors associated with enablers to adherence that are consistent with the extant literature. These include having good information about ARF [26, 35], good pain management techniques for injections [21, 24] and support from whānau and friends [29, 31]. Two relational/individual findings were relatively unique to this study. Participants noted that having negative interactions with DNs was a barrier to adherence more so than positive relationships was an enabler. These findings are consistent with literature that shows the negative interaction with social networks and healthcare providers has more impact on health behaviour than positive interactions do [41]. Further, participants who expressed personal responsibility for their health (e.g., taking their own initiative to research ARF) tended to have high levels of adherence to the injections, as did those with a greater understanding of their ARF diagnosis.

These individual and relational findings have consistency within the literature on adherence to medication for youth with chronic conditions [22, 23]. Some of the most common reasons identified for non-adherence in systematic reviews include family and peer support and information being important and yet not sufficient for adherence. In the current study, people who were adherent tended to have a good understanding of ARF and the need for injections and yet they attributed family support as a key reason for remaining adherent. Patients who did not have good information, lacked family support and had other resource barriers appeared to be at risk for intermittent or non-adherence.

It is not surprising that youth tended to focus on the individual and relational characteristics as those are more immediate to their everyday life. However, they did note some system and access issues in their responses. A key enabler for adherence was DNs that came to patients’ homes and workplace. This has been noted in previous research [24]. Further, patients noted that low income, especially as it relates to being able to communicate via mobile phone and getting time off work, is a barrier to adherence. Both of these findings have been noted in prior research although these points are not as prevalent in the literature as the individual characteristics [24, 25]. Further, these findings should be interpreted within the context of health inequities within New Zealand, particularly for Māori. While some system efforts such as the DN visits have enhanced adherence, there are still socio-structural barriers that contribute to non-adherence [9, 10]. The relative invisibility in the literature points to a bias of individual responsibility for medication adherence despite the need for continued health system changes.

We suggest that the health system has a key role for reducing inequities in ARF sequelae. Using a systems approach, we identify several recommendations that could be instituted in the health system and identified areas where multiple small improvements in improving secondary prophylaxis could be made. Nationally a RHF register would assist in the transfer of patients between DHB regions. This was found to be important because a number of patients on the prophylaxis regime are highly mobile and moved house a lot making it difficult to maintain effective communication. A national register would ensure that when patients left one area they could receive injections without a break as has been supported in prior literature [20, 28, 30].

Most patients described very positive interactions with their DNs. However this was not universal, and ensuring that DNs are well trained on health literacy could help increase adherence since negative interactions served as a barrier. Further, consistently high levels of individual understanding is partially dependent on a well organised and resourced health system, where health professionals know how to communicate key messages (i.e., effective health education) appropriate to the patient context, and ensure that patients and their family have the required high level of understanding [28]. However, the quality and amount of health information provided varies, with one study finding that Māori parents were given less information on patho-physiology of asthma than non-Māori parents [42]. Given this research, the lack of culturally appropriate, engaging and effective health messages is a potential systematic barrier than can be addressed through resources and staff training.

Pain is turning some patients away from injections; yet pain can be reduced through pain management [32, 34]. Although categorised as an individual factor, pain is also a health system and relational issue with DN technique and pain reduction tools having an influence on the patient’s experience of pain. For example, multiple patients stated that injections were much more painful when a different area from the normal was injected. Additionally, participants identified differences in pain management across the Waikato; some DNs offered nothing for the pain, some only suggested paracetamol and others offered all options currently available. Techniques for reducing injection site pain including vibrating cold packs, manual pressure and lidocaine [32, 34]. Lidocaine was just recently approved for use by the Waikato DHB and should reduce this particular barrier provided it is routinely used by the DNs.

Finally, reorientation of healthcare systems in the use of technology (e.g., mobile phones and apps) as a communication tool may provide a safe and easy avenue for patients to respond to nurses in their assessment and preparation for injections by asking patients which pain management strategies they would like. This technology is preferred by youth and young adults in general and specifically around health messaging [43, 44]. Youth can also follow up and assess the effectiveness of their interventions by assessing how the pain was and how long it lasted for, again in an easier format to answer over text. However, lack of phone credit, while also an individual issue, is something that can be addressed in the health system.

This study has several limitations. First, it is based on perceptual data rather an objective measurement of enablers and barriers. Such information is valuable to understand patient perspectives and context although cannot be used as specific predictors of adherence. Further, we did not include interviews with DNs or other members within the health system. Their viewpoints would be valuable to understanding the overall context. Despite these limitations, this study does provide insight into the numerous factors that collectively impact adherence to secondary prophylaxis for youth with ARF or RHD.

## Conclusion

In conclusion, many travel, cost and system barriers to RHF secondary prophylaxis have been reduced in the Waikato DHB region. For example, DNs travelling to patients to give injections saves patients time, stress and financial strain. Further, the register helps to track and communicate with patients. Adherence with long term medications for youth with chronic conditions is moderate – often sitting at around 50% adherence [22, 23]. So the 70% adherence rate for the Waikato programme, although not as high as it needs to be, is still higher than what it may have been without all the work done to date. We have identified several health system changes to further improve adherence rates and contribute toward removing the burden of illness and death due to RHD from New Zealand’s health system, communities, families and individuals.
